# PTH_1-34_ improves bone healing by promoting angiogenesis and facilitating MSCs migration and differentiation in a stabilized fracture mouse model

**DOI:** 10.1371/journal.pone.0226163

**Published:** 2019-12-10

**Authors:** Xin Jiang, Cuidi Xu, Hongli Shi, Qun Cheng

**Affiliations:** Department of Osteoporosis and Bone Disease, Huadong Hospital Affiliated to Fudan University, Research Section of Geriatric Metabolic Bone Disease, Shanghai Geriatric Institute, Shanghai, China; Università degli Studi della Campania, ITALY

## Abstract

**Objective:**

PTH_1-34_ (parathyroid hormone 1–34) is the only clinical drug to promote osteogenesis. MSCs (mesenchymal stem cells) have multidirectional differentiation potential and are closely related to fracture healing. This study was to explore the effects of PTH_1-34_ on proliferation and differentiation of endothelial cells and MSCs in vitro, and on angiogenesis, and MSCs migration during fracture healing in vivo.

**Methods:**

Mice with stabilized fracture were assigned to 4 groups: CON, PTH (PTH_1-34_ 40 μg/kg/day), MSC (transplanted with 10^5^ MSCs), PTH+MSCs. Mice were sacrificed 14 days after fracture, and callus tissues were harvested for microCT scan and immunohistochemistry analysis. The effects of PTH_1-34_ on angiogenesis, and MSCs differentiation and migration were assessed by wound healing, tube formation and immunofluorescence staining.

**Results:**

Treatment with either PTH_1-34_, or MSCs promoted bone healing and vascular formation in fracture callus. The callus bone mass, bone volume, and bone mineral density were all greater in PTH and/or MSC groups than they were in CON (p<0.05). PTH_1-34_ increased small vessels formation (diameter ≤50μm), whereas MSCs increased the large ones (diameter >50μm). Expression of CD31 within calluses and trabecular bones were significantly higher in PTH_1-34_ treated group than that of not (p<0.05). Expression of CD31, VEGFR, VEGFR2, and vWF was upregulated, and wound healing and tube formation were increased in MSCs treated with PTH_1-34_ compared to that of control.

**Conclusions:**

PTH_1-34_ improved the proliferation and differentiation of endothelial cells and MSCs, enhancing migration of MSCs to bone callus to promote angiogenesis and osteogenesis, and facilitating fracture healing.

## Introduction

Delayed healing and nonunion of fractures (collectively referred to as DNFs) are enormous burdens on patients and health care systems. Recent data specific to open long-bone fractures indicated that 17% had developed nonunion, and another 8% exhibited delayed union[1]. In the context of delayed/nonunion, fragile fractures in osteoporotic populations are associated with significant patient morbidity, loss of productivity, decreased quality of life, and extensive health-care utilization[2–3]. Healing of bone defects is a dynamic progenitor cell-driven tissue morphogenetic process that requires coordinated osteogenesis and angiogenesis at the site of the repair[4–5]. The regulatory pathways for the acceleration of fracture healing remain unclear[6]. Mesenchymal stem cells (MSCs) possess not only the ability to differentiate into osseous lineages such as osteoblasts, osteocytes and chondrocytes, etc. but have the potential to transdifferentiate into certain cell lineages: endothelial cells[7–9]. Recent studies both in vitro and in vivo utilizing animal models clearly identified bone marrow-derived MSCs as important contributors to the formation of new blood vessels in adults[10] in a process known as angiogenesis. Angiogenesis is a normal response to injury, and some angiogenic factors may have particular significance in bone growth and repair. Recent advances in identifying and cloning the many factors associated with angiogenic activity have renewed the interest in the possibility that the repair of difficult fractures can be enhanced. Some studies have confirmed an increase in blood flow and vascularity during the repair of fractured bone[11–12].

PTH_1-34_ is the first bone anabolic agent approved by the US Food and Drug Administration for the treatment of osteoporosis. Systemic PTH_1-34_ deficiency in mice is associated with impaired fracture healing[13], and exogenous PTH and PTH fragments can increase fracture callus density and promote early chondrogenesis and osteogenesis in fracture models[14], which may have favorable implications for the treatment of DNFs. At present, the mechanisms by which PTH_1-34_ promotes fracture healing need to be elucidated; however, stimulation of angiogenesis may be one such mechanism[15]. Recent studies have suggested that PTH_1-34_ treatment may affect mesenchymal stem cells from bone marrow to migrate into bloodstream[16].

In this study, we established a stabilized fracture mouse model that demonstrates a complete spatial pattern of revascularization in a stabilized fracture[17]. This study was designed specifically to prove the synergistic effect of PTH_1-34_ with transplanted MSCs to accelerate fracture healing via the angiogenesis pathway in the following ways: a) PTH_1-34_ enhances tube formation in bone calluses; b) PTH_1-34_ enhances the proliferation and biological function of endothelial cells in vitro; and c) PTH_1-34_ stimulates MSCs recruitment and homing to fracture sites, which promotes osteogenesis and angiogenesis.

## Materials and methods

### Experimental design and stabilized femur fracture model establishment

**Fig 1:**
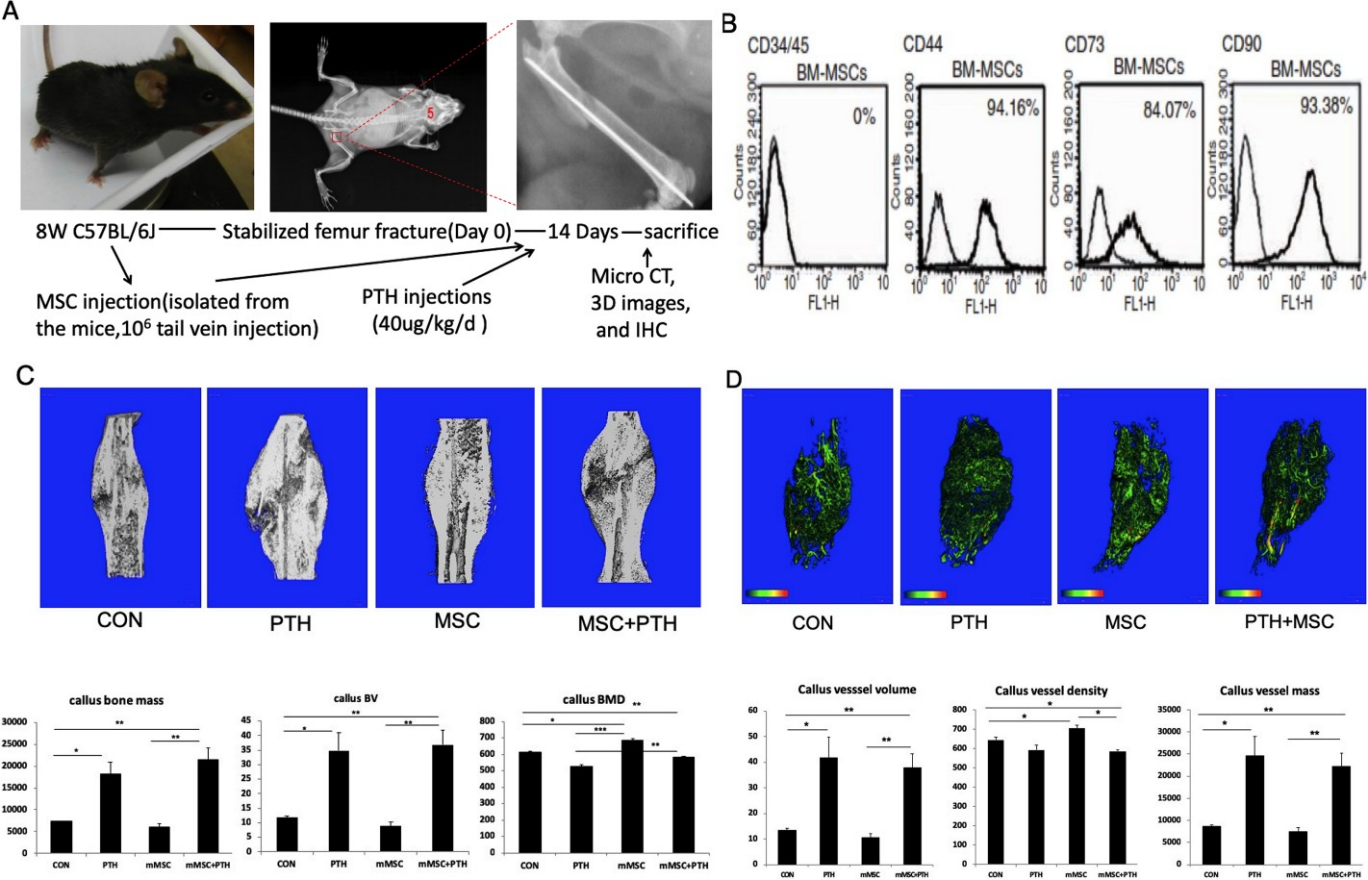
Experimental design and model establishment. (A) Stabilized femur fractures were produced in 6-week-old C57BL/6 female mice. Mice were divided into 4 groups: The control group was treated by subcutaneous injection of the vehicle (BSA) daily; the PTH group was treated with subcutaneous injection of 40 μg/kg/day of PTH (1–34); the PTH+MSC group and the MSC group were treated by injecting BM-MSCs systemically in combination with or without PTH. After 14 days of treatment, bone regeneration and neovascularization were monitored using μCT, and the samples were harvested for micro CT analysis, 3D images capture, and immunohistochemistry analysis. (B) We isolated and obtained an MSC population in which >90% of cells expressed the specific MSC markers CD90 and CD44 and 84.07% of the CD73 marker. Furthermore, after immunodepletion, the MSCs were negative for CD45 (0.9±0.5%). (C) Results from the μCT analysis and quantitative analysis of bone formation in fracture calluses was performed, and the callus bone mass, callus bone volume and callus bone mineral density were calculated. (D) The blood vessels detected by microCT were quantified in decalcified calluses. Data are presented as the mean +/− SEM (n = 12) for the groups. Student’s t test for two groups comparisons was performed; *P < 0.05, **P < 0.01, and ***P < 0.001.

All the procedures described in this study were approved by the animal care committee of Fudan University (Project NO.2014K004). 6-week-old female C57BL/6J mice from Shanghai Model Organisms Center.Inc were performed by intramedullar fixation using a 0.25mm stainless steel pin inserted through the patellar tendon inside the medullar canal of the femur. Bupremorphine (0.5 mg/kg) was administered subcutaneously as pain control. Postoperatively, the animals were randomly divided into four groups (n = 12 per group): (1) CON, mice were administrated with BSA; (2) PTH_1-34_, mice were administrated with PTH_1-34_ (Genekey Biotech, China)40ug/kg/d;(3)BM-MSCs transplatation, mice were administrated with BM-MSCs isolated from C57BL/6J mice 10^5^ tail vein injection; (4) PTH+BM-MSCs transplatation, mice were administrated with PTH 40ug/kg/d subcutaneously and BM-MSCs 10^5^ tail vein injection. Mice were sacrificed under deep anesthesia by exsanguination 14 days after fracture. The experimental procedure is shown in Fig 1A. Bone regeneration of femur injury sites was quantified by using in vivo microCT; angiogenesis was validated by performing microCT scan after perfusing with microfilm, 3D images and immunohistochemical analyses. The angiogenic differentiation of MSCs were analyzed by immunohistochemistry, immunofluorescence and tube-formation assays. MSC migration was observed by scratch wound healing assays. The effects of PTH on vascular cells was measured by qPCR, western blot, CCk-8, and tube-formation assay.

### Isolation and culture conditions for mouse MSCs and HUVEC

Bone marrow were dissected from mice femurs and filtered through 70*μ*m cell strainer, collected by 300×g for 10 min, and suspended in Dulbecco's modified Eagle's medium containing 1500 mg/l D-glucose supplemented with 20% fetal bovine serum (Biological Industries), 1% penicillin/streptomycin (P/S; Biological Industries), and 1% glutamine. Cells were seeded in the 25cm^2^ flask at a density of 1.6×10^6^ cells/cm^2^ and incubated at 37°C with 5% CO_2_. Bone marrow nucleated plasticadhering cells were expanded for 7–10 days without passaging. HUVECs were purchased from the American Type Culture Collection (ATCC, USA) and grown in Endothelial Cell Medium (1001, ScienCell, USA) at 37°C with 5% CO_2_.

### Characterization of cell surface markers

MSCs at 80% to 90% confluence were trypsinized and collected by centrifugation. Cells were incubated with antibodies against CD34/45 (MAB6518, R&D systems), CD44 (FAB6127G, R&D systems), CD73 (Larodan AB, Malmo, Sweden) and CD90 (FAB7335R, R&D systems) at 4°C for 10 min in the dark. Expression of the above surface markers was determined with flow cytometer (Canto II, Becton, Dickinson and Company).

### Quantification of new bone formation and vascularity using μCT analysis

Analysis of bone volume and vascular networks was performed by scanning the femurs in a CT imaging system (eXplore; GE HealthCare). Microfil MV-122 (Flowtech) contrast media was perfused through the heart along with 4% paraformaldehyde. After perfusion, the grafted femur was removed and scanned using a μCT imaging system to image bone volume. After complete decalcification by 10% EDTA solution, the samples were scanned again to image vascularization at the defect region.

### Immunohistochemical (IHC) analyses for evaluating angiogenesis in vivo

The slices were incubated with a 1/100 dilution of an anti-VEGF antibody (ab46154, Abcam) and an anti-CD31/PECAM-1 antibody (NB100-2284, Novus).The localization of CD31-and VEGF-positive cells were observed by a light microscope.

### Immunofluorescence (IF) analysis

MSCs cultured as above described were seeded on cover slips in 6-well plates. Briefly, After fixing and blocking, cells were labeled at 4°C overnight in the presence of an antibody directed against vWF (Dako Cytomation, Denmark),VEGFR1 (SigmaAldrich),VEGFR2 (abcam), and CD31 (abcam), respectively. Nuclei were labeled by adding 40-6-diamidino-2-phenylindole(Invitrogen,USA). Cells were photographed under a fluorescent microscope (Nikon, Japan).

### Tube formation assay

Geltrex® Matrix (Life Technologies) was added to 24-well plates and incubated at 37°C for 1 h. 1×10^4^ cells were plated and treated with conditioned medium containing 10 nM PTH1-34 for HUVEC or 100 nM for BM-MSC or not. The cytoskeleton were stained with Calcein, AM(Invitrogen).At different time points(1.5h, 3h,6 h for HUVEC and 20h for MSC), the formation of tube-like networks were captured using a fluorescent microscope. Three random microscopic fields were quantified using Image J software.

### RT-qPCR

Total RNA was extracted using TRIzol^®^ Reagent (Invitrogen) and PCR amplification was performed using an Applied Biosystems plus Detection System. Specific primers are synthesized by Sangon Biotech and listed in S1 Table. Expression level of target genes were normalized to GAPDH using the comparative 2^-ΔΔCt^ method.

### Western blot analysis

Equal amounts of total protein extracts were separated by SDS-PAGE, and transferred onto a PVDF membrane (Millipore, USA). After blocking, membranes were incubated with primary antibodies against VEGF, COX-2 (ab179800, Abcam) (,vWF, VEGFR1, VEGFR2, and CD31 respectively overnight at 4°C. The membranes were incubated with corresponding secondary antibodies from Cell Signaling and scanned using an ECL western blotting detection system (Bio-Rad, USA).

### Scratch wound healing assay

The BM-MSC monolayer was serum starved overnight and then scraped with a yellow pipette tip to generate scratch wounds. Cells were incubated for 24 h with conditioned medium containing 100 nM PTH_1-34_. Time lapse images were captured at 0, 2h, and 6h time points in the same position using a Nikon microscope. Three random microscopic fields in each sample were quantified by image pro plus software.

### Statistical analysis

All data represent at least three separate experiments. Values in vitro are expressed as mean±S.D. and in vivo are expressed as mean ± SEMs. Significant differences between two groups were determined by Student’s t test. Statistical significance is displayed as *P < 0.05, **P < 0.01 or ***P < 0.001.

## Results

### Increased bone healing after PTH_1-34_ and/or MSC treatment with increased number of vascular vessels in the stabilized femur fracture model in mice (Fig 1).

The healing of the bone fracture was evaluated by bone mass and vessel formation at the site of fracture callus with micro-CT at 14 days following surgery (Fig 1). The callus bone mass and bone volume were higher with PTH_1-34_ treatment compared with those not, regardless of whether or not the MSCs were administered (p<0.05) (Fig 1C). However, the callus BMD in MSC and PTH+MSC group was stronger than that in control and PTH group (p<0.05), suggesting that PTH_1-34_ promoted bone mass and increased bone volume, but calluses did not reach full mineralization at 14 days. MSCs promoted mineralization; however, they had no effect on bone mass or bone volume.

The vascular network in calluses at fracture sites was visualized after tissue decalcification 2 weeks to enable opacification with a contrast agent Microfil (Fig 1D) and then quantified and analyzed by micro-CT scanning. Micro-CT results showed infrequent vessels in CON group. More vessel mass and vessel volume were observed in callus with PTH treated group than those without PTH treated group (p<0.05) (Fig 1D), which was similar to that of callus bone mass and bone volume, suggesting that osteogenesis and angiogenesis are closely related to each other in bone callus.

### PTH1-34 treatment increased the formation of small vessels (diameter ≤50 μm), whereas MSCs treatment increased the formation of large vessels (diameter > 50 μm) (Fig 2)

**Fig 2:**
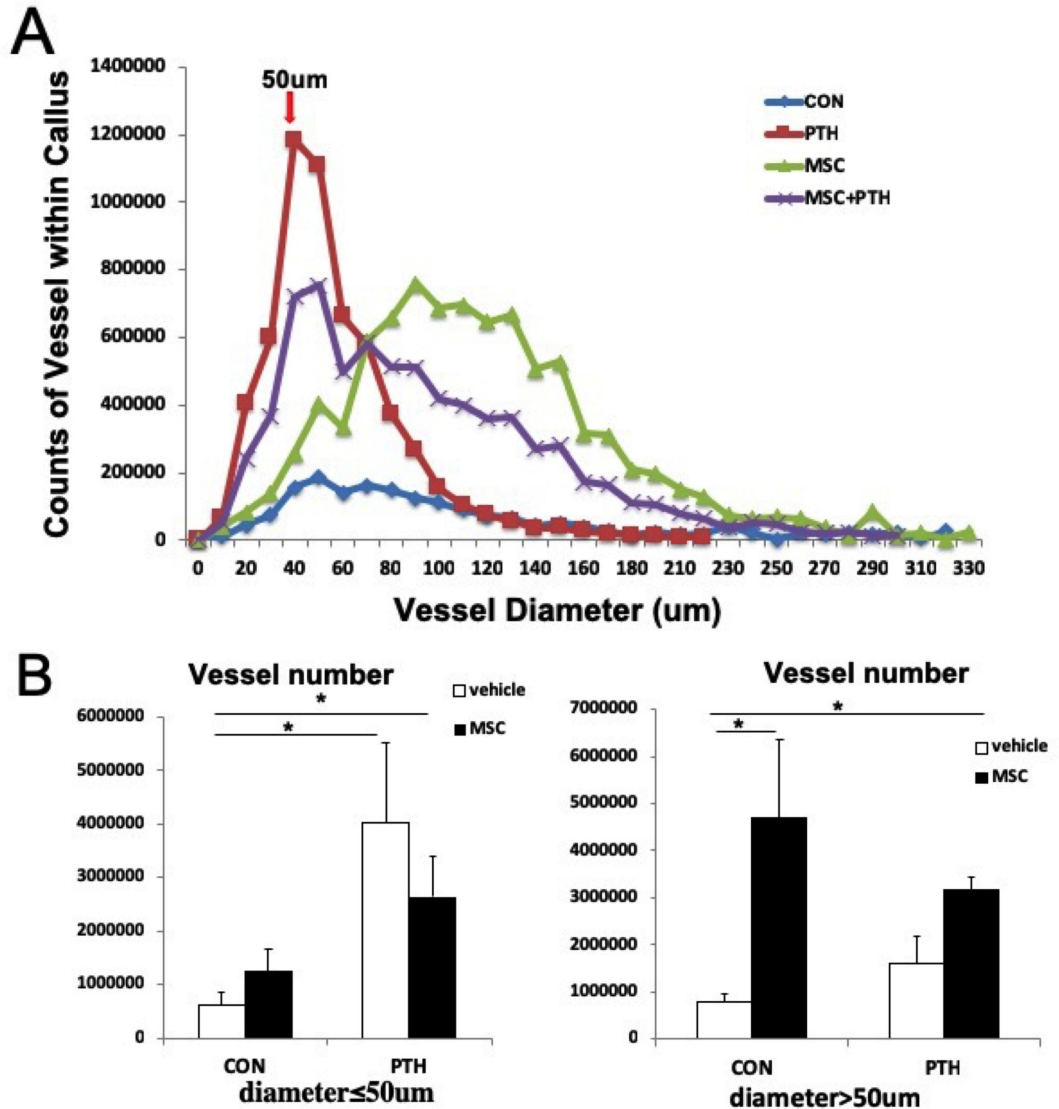
PTH promotes small vessel formation, whereas MSC promotes large vessel formation in calluses. (A) Vessel numbers and vessel diameter distribution as determined by micro CT scan in calluses in vivo. (B) Quantitative analysis of the vessel numbers in calluses. Student’s t test for two groups comparisons was performed; *P < 0.05.

Interestingly, we found that the newly formed vessels mainly consisted of microvessels (diameter ≤ 50 μm) for the group treated with PTH but not the groups without PTH exposure, as determined by diameter measurements of these vessels by micro-CT scanning. The number of the large callus vessels formed (diameter > 50 μm) increased significantly in the group treated with MSCs compared to the group that were not treated with MSCs (Fig 2).

We then analyzed VEGF expression at different microenvironment sites of bone callus (S1 Fig). VEGF expression was significantly higher in PTH-treated group than it was for other groups, and the trend in VEGF expression in mesenchyme was consistent with that in the newly formed microvessels (diameter ≤ 50 μm), which indicates that the expression of VEGF in microenvironment is correlated with vessel formation in bone calluses. Analysis of the visually detectable formation of microvascular networks indicated that PTH_1-34_ treatment promoted new tube formation and MSCs treatment enhanced vessel maturation in bone calluses.

### PTH_1-34_ promoted osteogenesis in bone calluses partly by its effect on vessel formation (Fig 3)

**Fig 3:**
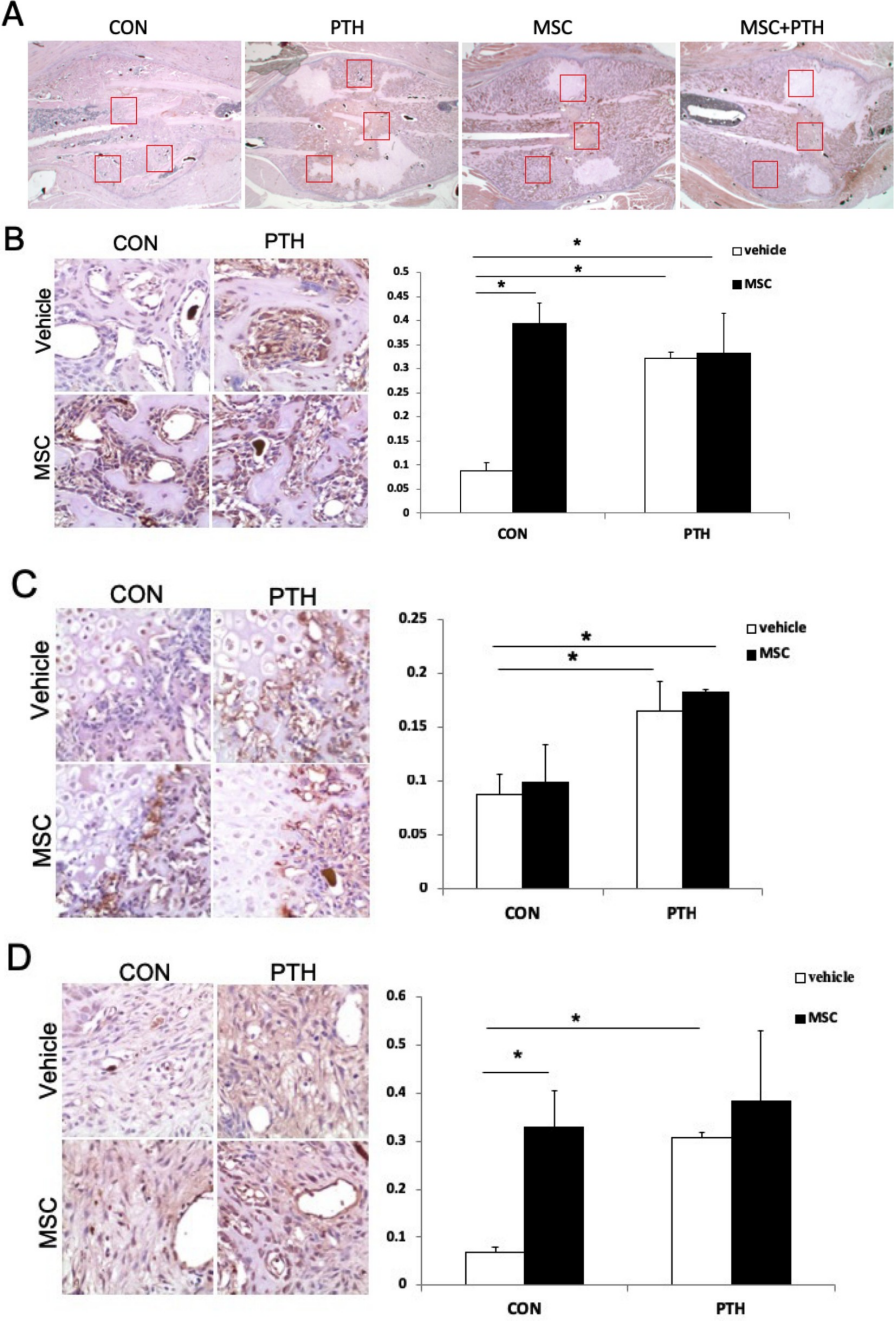
PTH promoted osteogenesis in bone calluses partly by its effect on vessel formation. (A) The expression of CD31 in calluses in the CON, MSC, PTH+/-MSC treatment groups. (B) Representative figure of CD31 expression and results from the quantitative analysis of trabecular bone. (C) Representative figure of CD31 expression and results from the quantitative analysis of the cartilage rim. (D) Representative figure of CD31 expression and results from the quantitative analysis in the mesenchyme. Micrographs of a representative tissue sections at different sites at 200× (A) and 400× (B-D). Student’s t test for two groups comparisons was performed; *P < 0.05.

The improved bone healing with angiogenesis promoted by PTH treatment was confirmed by immunohistochemical analysis (Fig 3). The expression of CD31 in trabecular bone, cartilage rim and mesenchyme in bone calluses was detected. Compared to control, the expression of CD31 was significantly increased in PTH-treated and MSC-treated groups in both the trabecular bone and the mesenchyme (p<0.05). However, it was also observed that CD31 expression in cartilage rim was increased significantly in the mice treated with PTH compared to those without. In addition, western blot result was shown that PTH treatment improved CD31 protein levels in MSCs during treatment with PTH (S2 Fig).CD31 is the specific marker of the “H” type vessel, which is the functional blood vessel mediating and inducing osteogenesis in bone marrow, suggesting that PTH_1-34_ may promote endochondral ossification in bone calluses during fracture healing.

### PTH_1-34_ directly increased the proliferation and physiological function of vascular endothelial cells in vitro (Fig 4)

**Fig 4:**
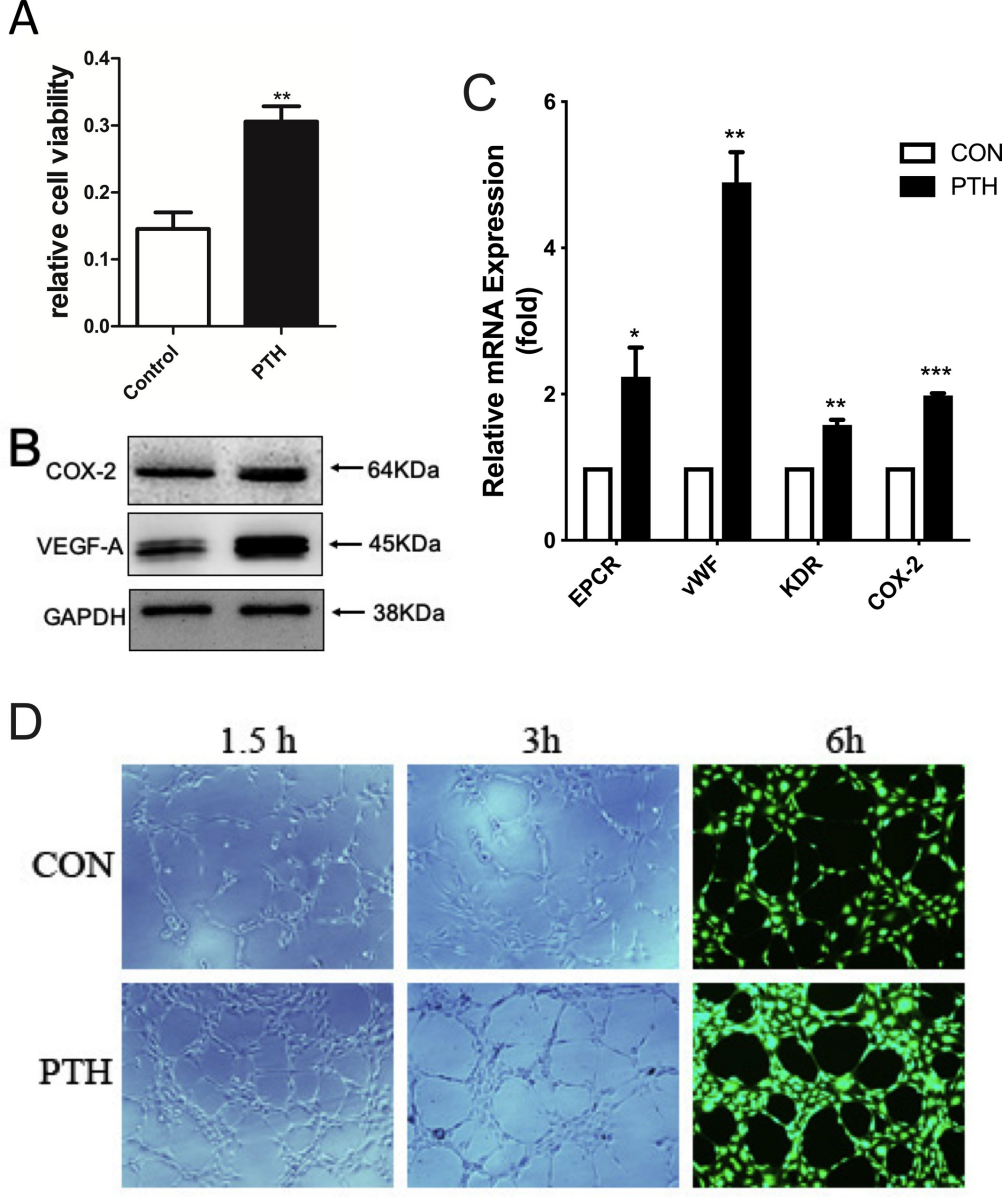
PTH enhances the proliferation and physiological function of endothelial cells in vitro. (A) HUVECs were treated with PTH or saline for 48 h (PTH treated 1 h every 24 h), and a CCK-8 assay was performed to determine the endothelial cell proliferative activity. (B) Western blot analysis for the expression of the VEGF and COX-2 protein levels. (C) qPCR for mRNA level analysis related to the function of endothelial cells after PTH treatment compared to that of the control. (D) The formation of tube-like networks was examined at 1.5 h, 3 h and 6 h by a tube formation assay. Representative micrographs obtained at 10× (D, scale bar, 50 μm). Student’s t test for two groups comparisons was performed; **P < 0.01, and ***P < 0.001.

To investigate the mechanisms of PTH_1-34_ enhancement of angiogenesis in process of bone healing, we first performed a CCK-8 assay on endothelial cells to assess cell proliferation after PTH_1-34_ treatment. The results showed significantly increased proliferation of endothelial cells treated with 10 nM PTH_1-34_ compared to that of CON (p<0.01) (Fig 4A).

QPCR assay showed that the expression of genes related to function of endothelial cells (EPCR, vWF and KDR) was significantly elevated by PTH treatment compared to that of CON (p<0.05) (Fig 4B). Western blot analyses showed that the expression levels of COX-2 and VEGF were both higher in endothelial cells treated with PTH compared to that of CON (Fig 4C). In addition, a significant increase in tube formation, including more tube numbers formed in less time, was observed in endothelial cells treated with 10 nM PTH1-34, according to tube formation assay (Fig 4D).These results suggested that PTH_1-34_ may directly promote angiogenesis by affecting the proliferation and differentiation of vascular endothelial cells during the healing of bone fractures.

### PTH_1-34_ indirectly enhanced angiogenesis by promoting migration and angiogenic differentiation of MSCs (Fig 5)

**Fig 5:**
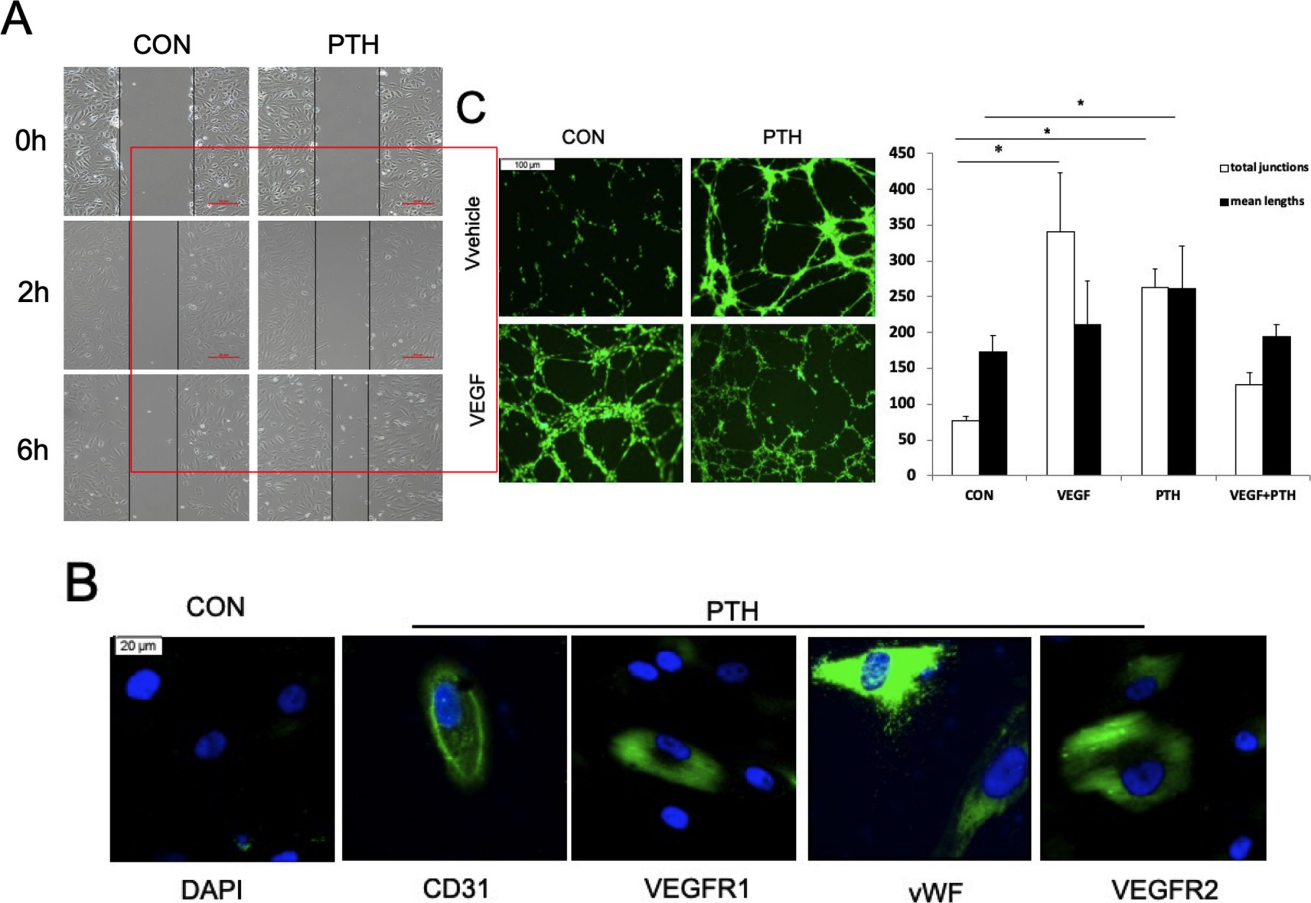
PTH promotes MSC migration and angiogenic differentiation in vitro. (A) Results from the scratch wound healing assay of MSCs after PTH treatment at different time points in vitro. (B) Immunofluorescence detection of vWF, CD31, VEGF1 and VEGFR2 expression in MSCs after 7 days of PTH treatment compared to that observed for the controls. (C) Tube-like network formation and quantitative analyses of the MSCs were examined after 20 h of PTH treatment. Representative micrographs obtained at 10× (A), 40× (B, scale bar, 10 μm) and 10× (C). Student’s t test for two groups comparisons was performed; *P < 0.05.

There is little information indicating the effect of PTH_1-34_ on the angiogenic microenvironment. In our study, PTH_1-34_ can recruited MSCs and significantly increased MSCs tube formation in vitro (Fig 5).

After incubation for 7 days or 14 days with PTH_1-34_, the protein expression of endothelial cell markers such as CD31, VEGFR1, VEGFR2, and vWF in MSCs was upregulated, according to immunofluorescence staining results (Fig 5B) and western blot results (S2 Fig). On the other hand, PTH_1-34_ also promoted wound healing and tube formation of MSCs in vitro (Fig 5A and 5C). These results confirmed that PTH_1-34_ promoted initial angiogenesis indirectly by improving MSCs migration and differentiation into endothelial-like cells.

## Discussion

Repair of bone defects requires recapitulation of signaling cascades in vivo, including those related to osteogenesis, chondrogenesis and angiogenesis, in an orchestrated spatiotemporal manner. Among other processes, angiogenesis occupies a central role in the whole process of bone regeneration after fracture[18–19]. VEGF is the most important factor with a significant role in both vasculogenesis (vasculature formation de novo) and angiogenesis (vessel formation from existing vasculature), but currently, only a limited number of studies have utilized VEGF for regeneration of bone defects in vivo[20]. Our study showed that femur-stabilized bone fractures generated in healthy mice were efficiently repaired following injection of PTH_1-34_or/and MSCs via promoting angiogenesis and osteogenesis. Although the ultimate fates of PTH, MSC, VEGF and other factors related to fracture healing are unknown, we demonstrate, for the first time, that PTH_1-34_ improves bone healing by directly promoting angiogenesis and indirectly affecting MSCs migration and angiogenic differentiation in a mouse stabilized fracture model, and this healing was correlated with CD31 expression, which provide clues as to the attributes of therapeutic agents that may someday prevent or treat DNFs.

In previous studies[21–22], researchers did not investigate the capacity of MSCs to induce bone regeneration in an injury model but rather examined their ability to augment intact bone structures. Here, we showed that the PTH_1-34_ treatment resulted in a significant enhancement in MSC migration in vitro, which is consistent with previously made conclusions[23]. However, one intriguing finding is that VEGF-A can induce VEGF receptor-negative MSCs to migrate and proliferate[24]. The Results of IHC analysis of VEGF and tube formation assay showed that PTH_1-34_ and VEGF can induce MSCs differentiation into endothelial-like cells. These results support the hypothesis that administration of PTH_1-34_ enhanced angiogenesis indirectly via MSC endothelial differentiation during bone healing.

Our results demonstrate that PTH_1-34_ not only directly enhanced the differentiation and tube formation of MSCs in vitro but also increased vessel volumn and vessel mass in the bone fracture site in vivo. Surprisingly, most of new vascular network surrounding bone calluses comprised small vessel formation (diameter ≤ 50 μm) in both PTH and PTH+MSC treatment groups, as demonstrated by results of blood vessel size tests, whereas the effect of MSCs treatment gave rise to increase of large vessels formation (diameter > 50 μm). Is the finding related to the collection time of the specimens, or does this finding reflect the pattern of angiogenic MSCs differentiation process during fracture healing? Recent studies clearly identify bone marrow-derived MSCs as important contributors to the formation of new blood vessels in adults [25]. Whether MSCs differentiation leads to vascular cells fate, such as endothelial cells, vascular smooth muscle cells (SMCs) or other cells is not well established defined and remains a controversial notion. Epigenetic factors, in particular histone deacetylases (HDACs) are proved to play an important role on the process of MSCs transdifferentiated into endothelial cells. Evidences show that there is a cross-talk between glucocorticoid receptor (GR) and HDAC2 and HDACs silencing upregulate cardiac-specific gene expression of MSCs, providing new explanations and insights into the regulation of MSC transdifferentiation fate[26–27].

Moreover, MSCs incorporated into vessel walls were reported to have differentiated towards CD31-positive endothelial-like cells [28]. Previous studies have identified a new capillary subtype: “type H endothelium” CD31^+^ vessels in the murine skeletal system that can mediate growth of new bone vasculature, maintain perivascular osteoprogenitors and couple angiogenesis to osteogenesis[29–30]. In this study, the results from IHC, IF and western blot analyses showed that CD31 expression and angiogenic differentiation of MSCs were upregulated after treatment with PTH_1-34_. Moreover, IHC analysis on trabecular bone, cartilage, and mesenchyme showed that VEGF expression level was consistent with that of CD31, which indicated that VEGF is also related to angiogenic differentiation of MSCs. S. D. Robinder demonstrated that PTH_1-34_ can cause a significant increase in small vessel numbers and decrease large vessel formation[31]. Thus, we hypothesize that PTH_1-34_ promotes VEGF expression in MSCs that are differentiating into endothelial cell lines, especially towards CD31-positive endothelial-like cells. Our next experiment will focus on this hypothesis and more quantitative data will be accomplished to support it.

Interestingly, in our study, the western blot analysis showed that expression of COX-2 was consistent with that of VEGF, which was higher in HUVECs treated with PTH_1-34_ than in control. Previous studies have shown that COX-2 is associated with endochondral angiogenesis[32], and endochondral ossification[33]. Therefore, during fracture healing, osteoclasts take charge of bone turnover and express COX-2 to induce vascular formation in cartilage rim, which are rich in CD31 high expressed endothelial cells to mediate the angiogenesis to osteogenesis conversion, however, this process is facilitated by PTH. The mechanism of COX-2 regulation of angiogenesis and the relationship of COX-2, VEGF and CD31 with fracture healing remains to be further explored.

The limitations of this study include the following: 1) this research indicates that PTH_1-34_ promotes migration and endothelial differentiation of MSCs, but there is no direct evidence to show the order of these processes; 2) the mechanisms of PTH_1-34_ promotion of the MSCs migration and endothelial differentiation have not been fully explored; however, we think it very likely that the mechanism depends on VEGF. In addition, to further explain these questions, more quantitative data are required, which could be obtained by sensitive and strict methods, such as RNAi and overexpression technologies or even VEGF-knockout animals.

In conclusion, present study confirmed that PTH_1-34_ enhanced osteogenesis by promoting angiogenesis as a result of the enhanced proliferation and function of endothelial cells, and the increased migration and endothelial differentiation of MSCs to promote fracture healing. This study also provides evidence that PTH_1-34_ could be attractive for future therapies designed to treat fractures in elderly osteoporosis patients and even nonunion fractures in clinical settings.

## Supporting information

S1 Fig PTH and/or MSCs treatment increased vascular endothelial growth factor in callus: IHC Assay. (A) IHC assay of VEGF expression in callus among MSC, PTH+/-MSC treatment groups. (B) IHC assay of VEGF expression and quantitative analysis in trabecular bone. (C) IHC assay of VEGF expression and quantitative analysis in the cartilage. (D) IHC assay of VEGF expression and quantitative analysis in mesenchyme. Micrographs of a representative tissue section at different site at 200× (A); and 400× (B-D). Student’s t test for two groups comparisons was preformed; *P < 0.05.
(TIFF)

S2 Fig vWF, CD31, VEGF1 and VEGFR2 protein levels in MSCs during treatment with PTH. Whole cell lysates were prepared from MSCs treated with 10 nM PTH1-34. Immunoblot analysis was performed for vWF, CD31, VEGF1 and VEGFR2 protein expression. β-actin was used as control for protein loading.
(TIF)

S1 Raw Images The original uncropped and unadjusted images relating to Fig 4C and S2 Fig. (TIF)

S1 Table Primer sequences for quantitative real-time PCR. (DOCX)
