# Supplementary figures and images for: PTH1-34 improves bone healing by promoting angiogenesis and facilitating MSCs migration and differentiation in a stabilized fracture mouse model
Source: PLoS One. 2019 Dec 10;14(12):e0226163. doi: 10.1371/journal.pone.0226163 (PMC6903750; doi:10.1371/journal.pone.0226163)

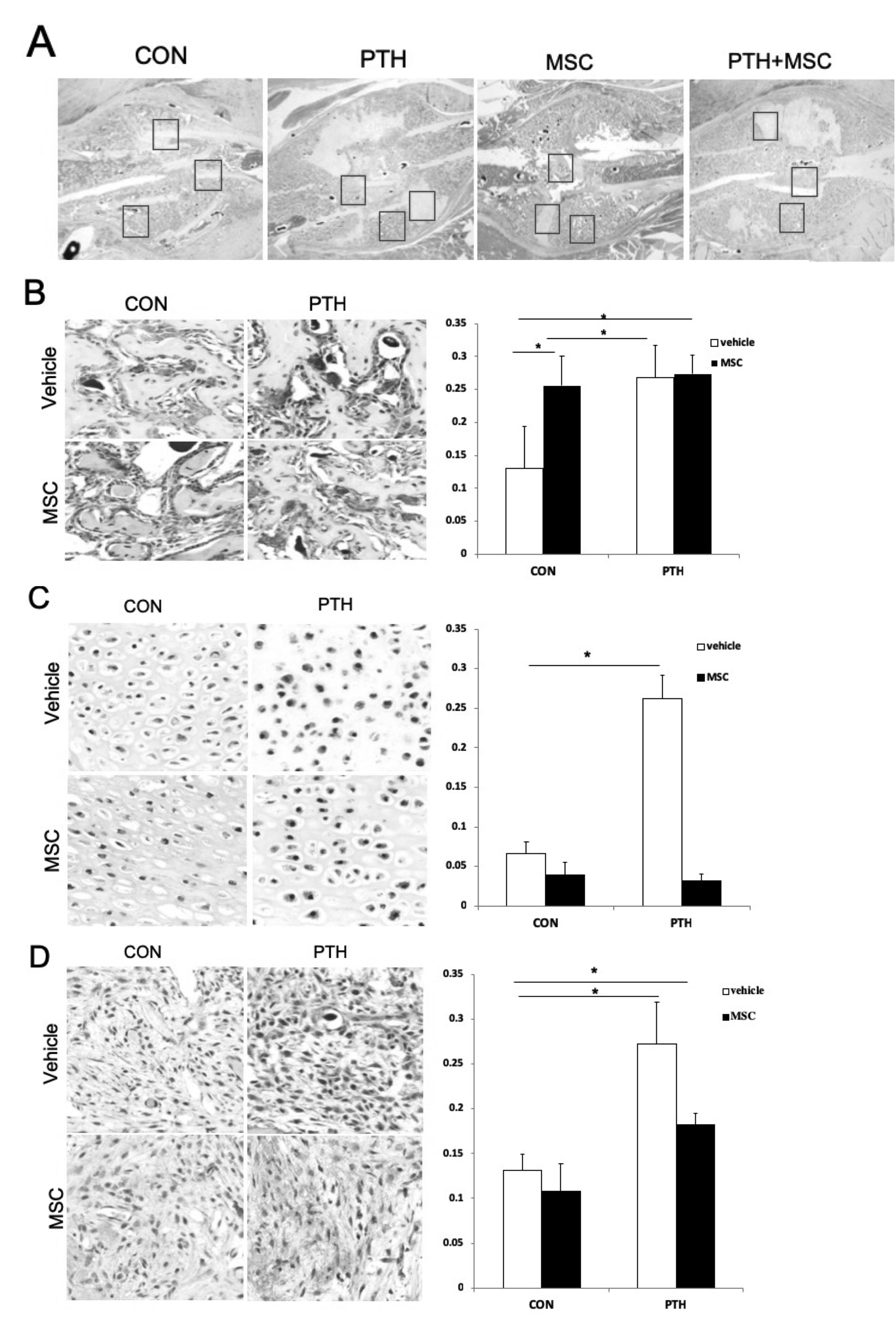

Supplement: S1 Fig — (A) IHC assay of VEGF expression in callus among MSC, PTH+/-MSC treatment groups. (B) IHC assay of VEGF expression and quantitative analysis in trabecular bone. (C) IHC assay of VEGF expression and quantitative analysis in the cartilage. (D) IHC assay of VEGF expression and quantitative analysis in mesenchyme. Micrographs of a representative tissue section at different site at 200× (A); and 400× (B-D). Student’s t test for two groups comparisons was preformed; *P < 0.05. (TIFF) [file pone.0226163.s001.tiff]

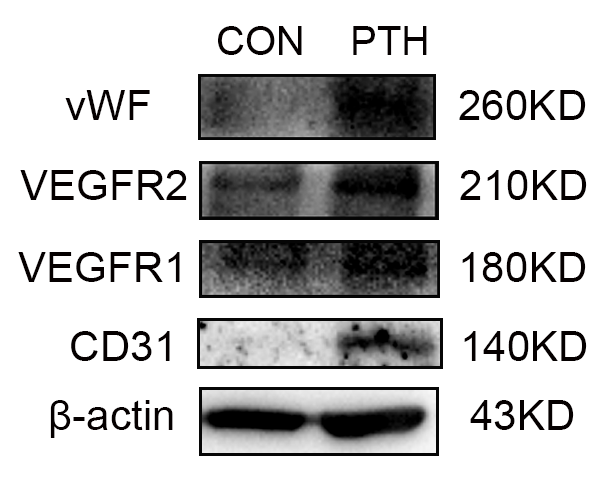

Supplement: S2 Fig — Whole cell lysates were prepared from MSCs treated with 10 nM PTH1-34. Immunoblot analysis was performed for vWF, CD31, VEGF1 and VEGFR2 protein expression. β-actin was used as control for protein loading. (TIF) [file pone.0226163.s002.tif]

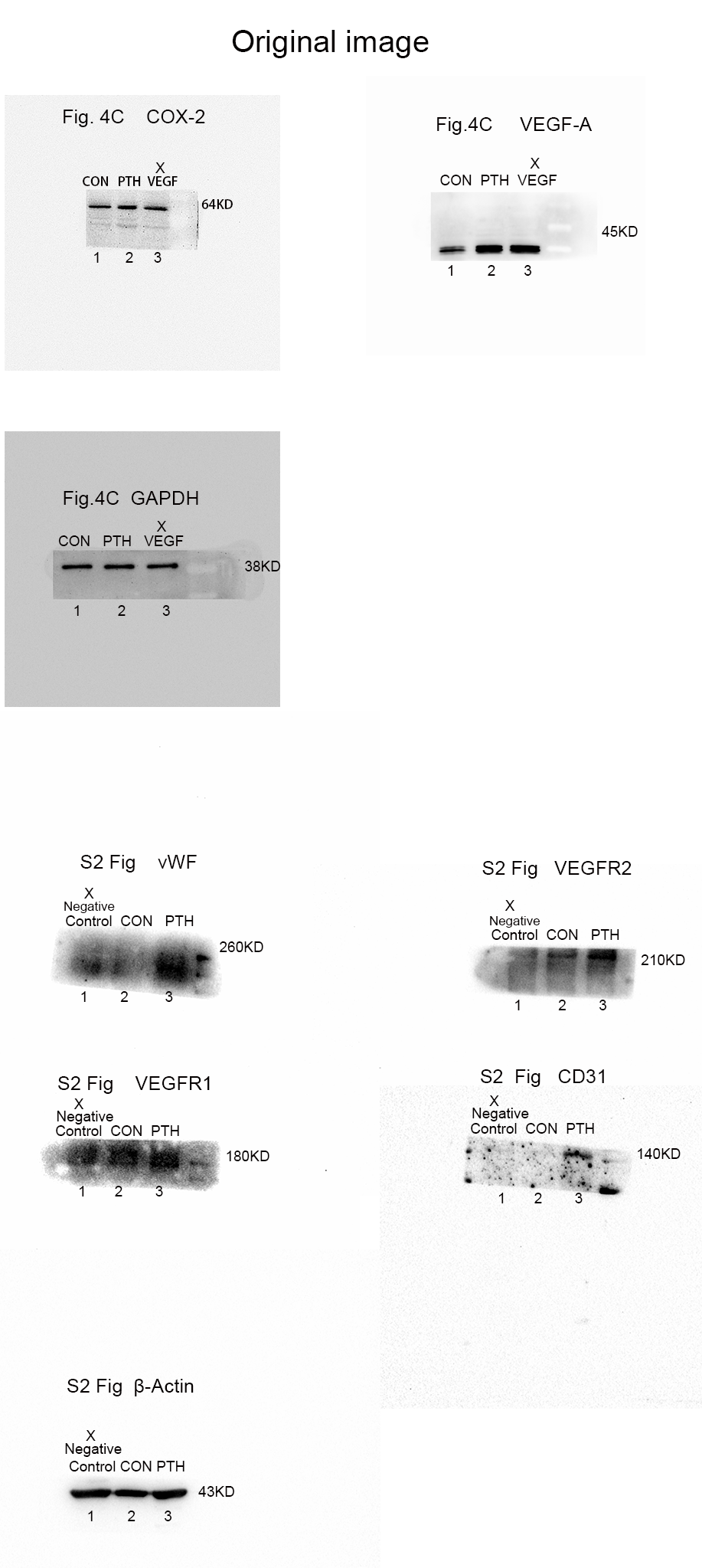

Supplement: S1 Raw Images — (TIF) [file pone.0226163.s003.tif]
