# Supplementary material for: PTH1-34 improves bone healing by promoting angiogenesis and facilitating MSCs migration and differentiation in a stabilized fracture mouse model
Source: PLoS One. 2019 Dec 10;14(12):e0226163. doi: 10.1371/journal.pone.0226163 (PMC6903750; doi:10.1371/journal.pone.0226163)
Supplement: S1 Table — (DOCX) [file pone.0226163.s004.docx]

**SUPPLEMENTARY TABLES**

Table 1. Primer sequences for quantitative real-time PCR

| Gene | Forward Primer | Reverse Primer |
| --- | --- | --- |
| GAPDH | AGGTCGGTGTGAACGGATTTG | TGTAGACCATGTAGTTGAGGTCA |
| EPCR | AATGCCTACAACCGGACTCG | AATGCCTACAACCGGACTCG |
| vWF | CTTCTGTACGCCTCAGCTATG | GCCGTTGTAATTCCCACACAAG |
| KDR | TTTGGCAAATACAACCCTTCAGA | GCAGAAGATACTGTCACCACC |
| COX-2 | TTCAACACACTCTATCACTGGC | AGAAGCGTTTGCGGTACTCAT |
